# Food allergy and anaphylaxis – 2058. Elucidation of a 31 Kda cross-reactive kidney bean

**DOI:** 10.1186/1939-4551-6-S1-P141

**Published:** 2013-04-23

**Authors:** Komarla Nagendra, Ramkrashan Kasera, Shakuntala Lavasa, Anand B Singh, Komarla Nagendra, Naveen Arora

**Affiliations:** 1 Bangaluru Allergy Centre, Bangalore, Bangalore, India; 2 Allergy and Immunology, Csir-Institute of Genomics and Integrative Biology, Delhi, India; 3 Uslavasa Medical & Research Center, Chandigarh, India; 4 Allergy and Allergen Research, Allergy and Immunology Lab, Institute of Genomics and Integrative Biology, Delhi University Campus Delhi, Delhi, India; 5 Allergy and Immunology Section, Institute of Genomics and Integrative Biology, Delhi, India

## Background

Legumes are rich source of proteins with significant nutritional advantages and may elicit IgE-mediated food allergy. Cross reactivity among legumes is very high because they are phylogenetically related. The present study was aimed to isolate and characterize a major allergenic protein of *Phaseolus vulgaris*and to study its cross reactivity.

## Methods

An allergenic protein of kidney bean was purified using anion exchange (Q Sepharose), gel filtration (Superdex 75) and reverse phase (C_18_) chromatography. Sera were collected from patients with marked positive skin reactions to kidney bean extract. The purified protein was characterized by immunobiochemical methods and identified by mass spectrometry.

## Results

Seventy five mg of allergen extract was loaded on to Q Sepharose column and adsorbed proteins were eluted using NaCl gradient. The eluted fractions with high intensity single band were pooled for further purification using Superdex 75 and C_18_column. In the major eluted peak a 31 kDa protein appeared as a single band on SDS-PAGE after silver staining and was recognized by kidney bean hypersensitive pooled patients’ sera on immunoblotting. Purified protein showed IgE binding to 88% of kidney bean hypersensitive patients’ sera by immunoblotting, thus indicating it to be a major allergen. The purified protein was potent and required 102 ng of homologous protein for 50% inhibition of IgE binding by ELISA whereas with crude extract required 976 ng. A total of 78 % kidney bean sensitive patients showed SPT positive reaction to purified 31 kDa allergen. These patients also showed significant histamine release with 10 ng each of extract and purified protein. Mass spectrometric analysis identified the purified protein as lectin (phytohemagglutinin). This protein was identified by PAS staining suggesting this protein as a glycoprotein. But no change in IgE binding was observed after periodate oxidation. Purified protein showed cross reactivity with peanut, black gram and pigeon pea and required 185, 228, and 1300 ng of protein(s), respectively for 50% inhibition.

## Conclusions

A 31 kDa major allergen of kidney bean was purified from *Phaseolus vulgaris*. Immunobiochemical characterization revealed its cross reactivity with peanut, black gram and pigeon pea.

